# rSjP40 suppresses hepatic stellate cell activation by promoting microRNA‐155 expression and inhibiting STAT5 and FOXO3a expression

**DOI:** 10.1111/jcmm.13819

**Published:** 2018-08-09

**Authors:** Dandan Zhu, Chunzhao Yang, Pei Shen, Liuting Chen, Jinling Chen, Xiaolei Sun, Lian Duan, Li Zhang, Jinhua Zhu, Yinong Duan

**Affiliations:** ^1^ Department of Pathogen Biology School of Medicine Nantong University Nantong China; ^2^ Laboratory Medicine Center Affiliated Hospital of Nantong University Nantong China; ^3^ Department of Medical Informatics School of Medicine Nantong University Nantong China

**Keywords:** hepatic fibrosis, hepatic stellate cells, microRNA‐155, rSjP40, *Schistosoma japonicum*

## Abstract

Activation of hepatic stellate cells (HSCs) is the central event of the evolution of hepatic fibrosis. Schistosomiasis is one of the pathogenic factors which could induce hepatic fibrosis. Previous studies have shown that recombinant *Schistosoma japonicum* egg antigen P40 (rSjP40) can inhibit the activation and proliferation of HSCs. MicroRNA‐155 is one of the multifunctional noncoding RNA, which is involved in a series of important biological processes including cell development, proliferation, differentiation and apoptosis. Here, we try to observe the role of microRNA‐155 in rSjP40‐inhibited HSC activation and explore its potential mechanisms. We found that microRNA‐155 was raised in rSjP40‐treated HSCs, and further studies have shown that rSjP40 enhanced microRNA‐155 expression by inhibiting STAT5 transcription. Up‐regulated microRNA‐155 can down‐regulate the expression of FOXO3a and then participate in rSjP40‐inhibited expression of α‐smooth muscle actin (α‐SMA) and collagen I. Furthermore, we observed microRNA‐155 inhibitor could partially restore the down‐regulation of FOXO3a, α‐SMA and collagen I expression in LX‐2 cells induced by rSjP40. Therefore, our research provides further insight into the mechanism by which rSjP40 could inhibit HSC activation via miR‐155.

## 1 INTRODUCTION

Hepatic fibrosis is a common precursor of many chronic liver diseases and a necessary stage for the development of chronic liver injury to cirrhosis. Studies have shown that the process of hepatic stellate cells (HSCs) turning from quiescent state to activation state plays a central role in the development of hepatic fibrosis.^1^, ^2^ Activated HSCs activate into myofibroblasts, resulting in deposition and unbalanced degradation of extracellular matrix (ECM).^3^, ^4^ Previous studies have shown that soluble egg antigens (SEA) from *Schistosoma japonicum* (*S. japonicum*) and *Schistosoma mansoni* (*S. mansoni*) can inhibit HSC proliferation and activation.^5^, ^6^ In our laboratory, our previous study showed that SEA of *Schistosoma japonicum* can inhibit the activation of HSCs and induce HSC apoptosis both in vitro and in vivo.^7^ As SEA is a complex mixture containing many antigens, and the egg antigen P40 of *S. japonicum* (SjP40) is the main component of SEA,^8^, ^9^, ^10^ our laboratory also focused on the effect of the recombinant SjP40 (rSjP40) on HSC activation. We found that rSjP40 could inhibit α‐smooth muscle actin (α‐SMA) and collagen I expression in transforming growth factor‐β1 (TGF‐β1)‐treated LX‐2 cells.^11^ Further study also confirmed that the expression of the p27 promoter was enhanced in HSCs through an E2F1‐dependent mechanism.^12^ Interestingly, rSjP40 could also promote HSC senescence and cell cycle arrest through the STAT3/p53/p21 pathway.^13^

MicroRNAs (miRNAs) are a class of noncoding small RNAs that are prevalent in animals and plants.^14^ miRNAs can regulate gene expression at the post‐transcriptional level by degrading mRNA or inhibiting mRNA translation. miRNAs are involved in a series of important biological processes, such as cell proliferation, differentiation and apoptosis and play an important role in the physiological and pathological process.^15^, ^16^ More and more studies have shown that miRNAs play an indispensable role in the progression of hepatic fibrosis. For example, miR‐145 inhibits the activation and proliferation of HSCs through regulating the Wnt/β‐catenin signalling pathway.^17^ miR‐30 exerts its inhibitory effect by inhibiting the TGF‐β signalling pathway,^18^ and miR‐15b and miR‐16 are reported to be required for the induction of HSC apoptosis by targeting Bcl‐2 through the caspase signalling.^19^ As a multifunctional miRNA, miR‐155 has been shown to involve in the regulation of various biological processes including infection, atherosclerosis, oncogenesis, inflammation and immunity.^20^, ^21^, ^22^, ^23^ Recently, miR‐155 has been reported to inhibit the activation of HSCs through the ERK1 pathway in activated HSCs and simultaneously inhibit the progression of epithelial‐mesenchymal transition (EMT) in cells.^24^ However, whether miR‐155 plays a role in the inhibition of HSC activation by rSjP40 has not been reported yet.

Therefore, in this study, we sought to explore the role of miR‐155 in the inhibition of HSC activation by rSjP40 and to explore its underlying molecular mechanisms.

## 2 MATERIALS AND METHODS

### 2.1 Cell culture and treatment

LX‐2 was a well‐characterized human HSCs’ line obtained from Nantong Third people's Hospital, and LX‐2 cells were cultured in Dulbecco's modified Eagle's Medium (DMEM, Gibco, Thermo Fisher, Waltham, MA, USA) supplemented with 10% of foetal bovine serum (FBS), in a humidified atmosphere containing 5% CO_2_ at 37°C. LX‐2 cells were seeded into 6‐well culture plates and treated with rSjP40 at the concentration of 20 μg/mL. rSjP40 was obtained as previously described.^11^

### 2.2 Bioinformatics analysis of miR‐155 promoter and construction of plasmids containing miR‐155 promoter sequence and dual‐luciferase reporter assay

The sequence of the miR‐155 promoter (−2000 bp to +200 bp), encompassing the ATG start site, was obtained from the National Center for Biotechnology Information (NCBI, http://www.ncbi.nlm.nih.gov/). Transcription factor binding sites were predicted using the PROMO (http://alggen.lsi.upc.es/cgi-bin/promo_v3/promo/promoinit.cgi?dirDB=TF_8.3) and UCSC network platform (http://genome.ucsc.edu/index.html). To construct miR‐155 promoter‐associated plasmids, the polymerase chain reaction (PCR) primers were designed as Table 1. Genomic DNA was extracted from LX‐2 cells according to instructions for the QIAamp^®^ DNA Micro Kit (Qiagen, Hilden, Germany). Promoter fragment of miR‐155 from −2000 bp to +200 bp was amplified from genomic DNA template, cloned into pGL3‐basic vector (Promega, Madison, WI, USA) and renamed as pGL3‐pro‐miR‐155.

**Table 1:** Primers used in this study

Primer	Sequence (5′→3′)	Purpose
pro‐miR‐155 F	CTGTTGTTGGTTGCTTAGCC	miR‐155 promoter
pro‐miR‐155 R	TATGTAGGAGTCAGTTGGAGGC	
FOXO3a F1	ACGGAATAGTTGGGACCACCT	Wild FOXO3a 3′UTR
FOXO3a R1	AGGCAGGAGACGTGTGACAATAC	
FOXO3a F2	ATAAAACTAGACTGGCATATAAATGTATAAATA	Mutant FOXO3a 3′UTR
FOXO3a R2	ATGCCAGTCTAGTTTTATGCAAAGAAAAGAGT	
STAT5(a) F	GAGTGCTCTAATCAGGCAATTCG	ChIP (−1383 bp to −1371 bp)
STAT5(a) R	GCCACCCCAGCCAATATAACT	
STAT5(b) F	TATGGGTAGAATAGTATGCCAGCA	ChIP (−1383 bp to −1371 bp)
STAT5(b) R	TTTCCCCCTTACAGGGTCCT	
STAT5(c) F	AACCACGATATTCTGCCCTGTC	ChIP (−989 bp to −977 bp)
STAT5(c) R	CATTCAAGTATGTGGTCTCCTGC	
STAT5(d) F	ACCACGATATTCTGCCCTGTC	ChIP (−989 bp to −977 bp)
STAT5(d) R	TATCATTCAAGTATGTGGTCTCCTG	

F, Forward; R, Reverse.

Reporter plasmids were transiently transfected into LX‐2 cells according to the manufacturer's instructions for FuGENE transfection reagent (Promega, Madison, WI, USA). After transfection for 24 hours, rSjP40 was added and LX‐2 cells were cultured for another 24 hours. Then, the cells were harvested for luciferase activity analysis on a luminometer following the instructions of the dual‐luciferase reporter assay system (Promega, Madison, WI, USA).

### 2.3 Construction and luciferase assay of 3′UTR of FOXO3a

The sequence of the FOXO3a 3′UTR was obtained from the National Center for Biotechnology Information (NCBI, http://www.ncbi.nlm.nih.gov/). The binding site for miR‐155 from the FOXO3a 3′UTR was predicted using the miRBase (http://microrna.sanger.ac.uk/) and TargetScan (http://www.targetscan.org/). To construct FOXO3a 3′UTR‐associated plasmids, the PCR primers were designed (Table 1). Genomic DNA was extracted from LX‐2 cells according to instructions for the QIAamp^®^ DNA Micro Kit (Qiagen, Hilden, Germany). The sequence of the FOXO3a 3′UTR was amplified from genomic DNA template, cloned into psiCHECK‐2 luciferase vector (Promega, Madison, WI, USA) and renamed as psiCHECK2‐FOXO3a. The predicted binding site of the wild‐type FOXO3a 3′UTR was AGCATTA, and the binding site of the mutant‐type FOXO3a 3′UTR was CTAGACT. For dual‐luciferase reporter assays, the wild‐type FOXO3a 3′UTR or mutant FOXO3a 3′UTR plasmids and miRNAs were cotransfected into LX‐2 cells using Lipofectamine 2000 (Invitrogen, Thermo Fisher, Waltham, MA, USA). After transfection for 48 hours, the cells were collected and luciferase activities were analysed by the dual‐luciferase assay kit (Promega, Madison, WI, USA).

### 2.4 Chromatin immunoprecipitation

Chromatin immunoprecipitation (ChIP) experiments were performed using SimpleChIP Kit (Cell Signaling Technology, Danvers, MA, USA). Anti‐STAT5 antibody was purchased from Cell Signaling Technology (USA) and was used to precipitate the DNA‐protein complex. Normal IgG provided in SimpleChIP Kit was used as the negative control. Purified DNA obtained from SimpleChIP Kit was then used as the template, and PCR was conducted using primers in Table 1, which were designed based on the different STAT5‐binding sites in the miR‐155 promoter.

### 2.5 Western blot

Total proteins were extracted from LX‐2 cells using standard methods. Protein concentration was quantified by Bradford method (Sangon, Shanghai, China). Protein samples were separated by SDS‐PAGE (8%‐12%), transferred onto PVDF membranes (Merck, Darmstadt, Germany) and blocked with 5% nonfat dry milk. Membranes were incubated with specific primary antibodies at 4°C overnight and then incubated with an appropriate second antibody at room temperature. A chemiluminescence (ECL) kit (Merck, Darmstadt, Germany) was used to detect target proteins. Protein bands were normalized to GAPDH, and protein expression was quantified by Image Lab of Bio‐Rad (Berkeley, California, USA).

### 2.6 Quantitative real‐time PCR

The miRNAs were extracted using RNAiso for Small RNA (TAKARA, Kyoto, Japan) and transcribed into cDNA as previously described.^25^ cDNA products were worked as the template for Quantitative real‐time PCR (RT‐qPCR) analysis with a SYBR Premix Ex Taq Kit (TAKARA, Kyoto, Japan) on the StepOnePlus Real‐Time PCR System (Applied Biosystems, Waltham, MA, USA). All samples were run in triplicate, and the relative expression levels were determined by normalization to U6.

### 2.7 Statistical analysis

The statistical significance of differences was determined by SPSS software using the methods of independent Student's t test or one‐way ANOVA (least significant difference, LSD). All data were harvested from at least three independent experiments and presented as the mean ± SEM. *P* < 0.05 was considered statistically significant.

## 3 RESULTS

### 3.1 The expression of miR‐155 in LX‐2 cells is enhanced by rSjP40

**Figure 1:**
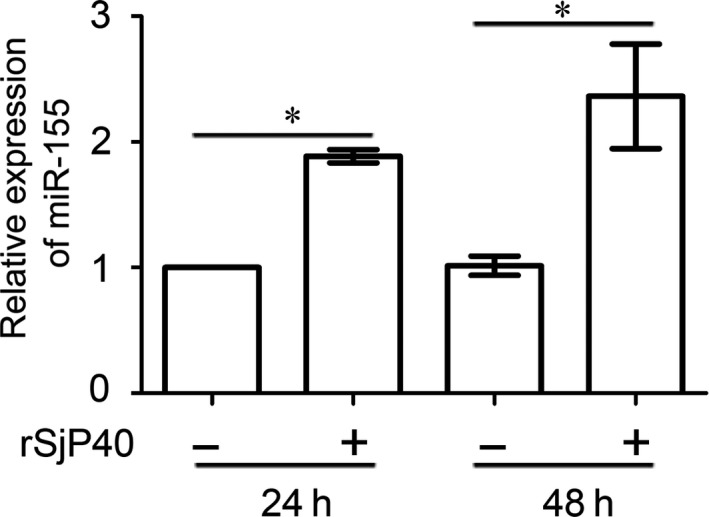
miR‐155 expression is up‐regulated in LX‐2 cells treated with rSjP40. The expression levels of miR‐155 in LX‐2 cells which were treated with rSjP40 at the concentration of 20 μg/mL for 24 or 48 h were detected by RT‐qPCR. *P < 0.05, compared to each untreated group. The data are presented as the mean ± SEM of at least three independent experiments

To confirm the expression of miR‐155 in inhibition of LX‐2 activation by rSjP40, RT‐qPCR was performed firstly and the results showed that miR‐155 expression was up‐regulated after LX‐2 cells were treated with rSjP40 (20 μg/mL) (Figure 1). This result is identical to that reported by Dai et al,^24^ who reported that the expression of miR‐155 was inhibited in TGF‐β1‐treated primary HSCs and activated HSC‐T6 cell line. Hence, we speculated that rSjP40 can inhibit HSC activation by promoting miR‐155 expression in LX‐2 cells.

### 3.2 FOXO3a is the potential target gene of miR‐155

**Figure 2:**
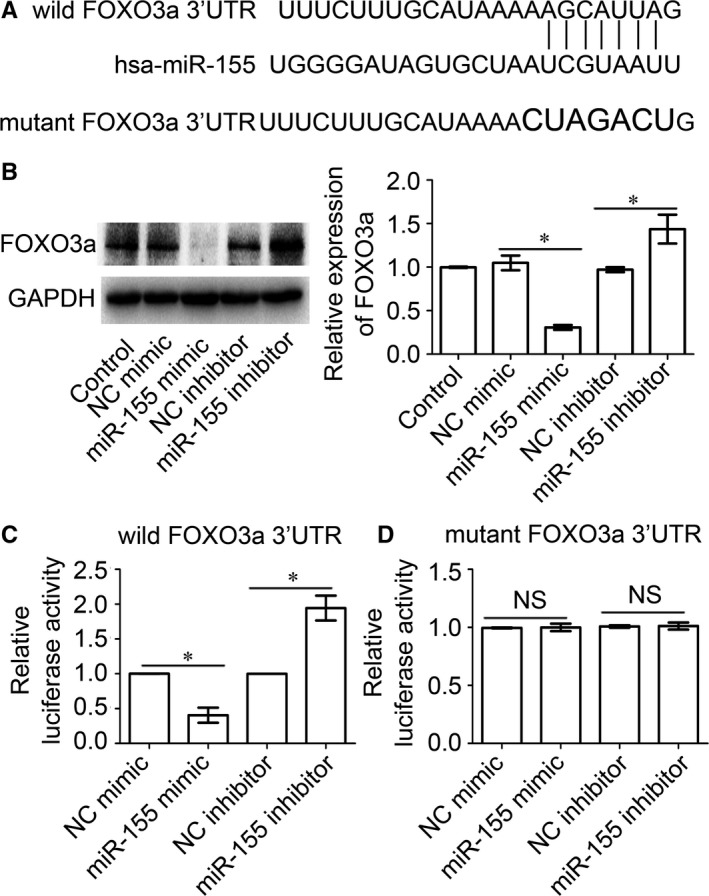
FOXO3a is the target gene of miR‐155. A, The wild and mutant binding sites of miR‐155 in FOXO3a 3′UTR sequences were shown. B, Expression levels of FOXO3a protein in LX‐2 cells transfected with miRNA‐155 mimic or inhibitor were determined using Western blot. *P < 0.05, compared to each NC group. C, Relative luciferase activities of wild FOXO3a 3′UTR in LX‐2 cells transfected with miRNA‐155 mimic or inhibitor were determined by dual‐luciferase reporter assay. *P < 0.05, compared to each NC group. D, Relative luciferase activities of mutant FOXO3a 3′UTR in LX‐2 cells transfected with miRNA‐155 mimic or inhibitor were determined by dual‐luciferase reporter assay. NS represents *P* > 0.05, compared to each NC group. All the data above are presented as the mean ± SEM of at least three independent experiments

Studies have shown that FOXO3a is targeted by miR‐155 in renal cell carcinoma^26^ and enterocyte.^27^ By software prediction, we also found that FOXO3a is a potential target for miR‐155 (Figure 2A). To identify the relationship between FOXO3a and miR‐155 in LX‐2 cells, miR‐155 mimic or inhibitor was transfected into LX‐2 cells, and then, FOXO3a expression level was detected. Western blot results showed that FOXO3a protein expression was decreased in LX‐2 cells transfected with miR‐155 mimic, while FOXO3a protein expression was increased in LX‐2 cells transfected with miR‐155 inhibitor (Figure 2B). To further confirm the direct effect of miR‐155 on FOXO3a, we constructed a wild‐type plasmid containing the FOXO3a 3′UTR sequence. Results from dual‐luciferase reporter assay showed that compared with the control group, the luciferase activity of 3′UTR of FOXO3a was decreased in the miR‐155 mimic transfected group and increased in miR‐155 inhibitor transfected group (Figure 2C). After the binding sites of the miR‐155 in FOXO3a 3′UTR sequences were mutated (Figure 2A), both the mimic and inhibitor could not regulate the activity of mutated FOXO3a 3′UTR plasmids (Figure 2D). The above data show that miR‐155 is directly combined with FOXO3a 3′UTR to regulate FOXO3a expression level in LX‐2 cells.

### 3.3 miR‐155 is involved in inhibiting the activation of LX‐2 cells by rSjP40 via FOXO3a

**Figure 3:**
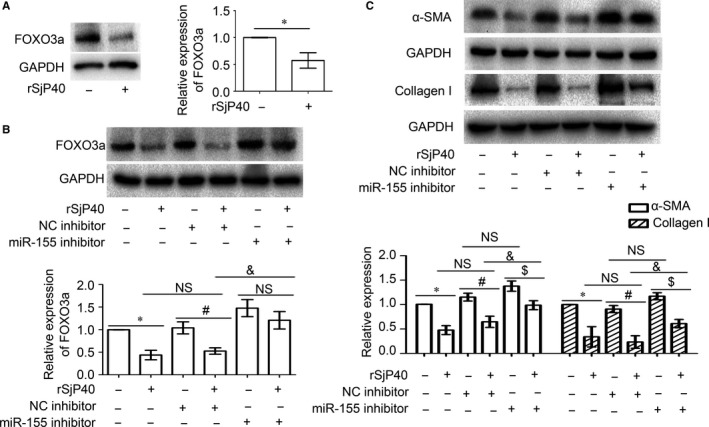
miR‐155 inhibitor can partially block down‐regulation of FOXO3a, α‐SMA, collagen I expression by rSjP40. A, The expression of FOXO3a protein levels in LX‐2 cells which were treated with rSjP40 (20 μg/mL) after 24 h was detected by Western blot. *P < 0.05, compared to untreated group. B, The expression levels of FOXO3a protein in LX‐2 cells which were transfected with miRNA‐155 inhibitor or NC inhibitor and treated with or without rSjP40 were detected by Western blot. C, The expression levels of α‐SMA and collagen I protein in LX‐2 cells which were transfected with miRNA‐155 inhibitor or NC inhibitor and treated with or without rSjP40 were detected by Western blot. The data are presented as the mean ± SEM of at least three independent experiments. *P < 0.05, compared to untreated group. ^#^ *P* < 0.05, compared to NC inhibitor + rSjP40− group. ^$^ *P* < 0.05, compared to miR‐155 inhibitor + rSjP40− group. ^&^ *P* < 0.05, compared to NC inhibitor + rSjP40 + group. NS represents *P* > 0.05 and shows that there is no significant statistical difference between the compared two groups

Our previous studies show that SEA induces the senescence of activated HSCs through the FOXO3a/SKP2/p27 pathway.^28^ As SjP40 is the main antigen of SEA, we further explore the role of miR‐155 and FOXO3a in the inhibition process of LX‐2 cell activation by rSjP40. Western blot results showed that FOXO3a protein expression was significantly inhibited in rSjP40‐treated LX‐2 cells (Figure 3A), and FOXO3a protein expression was up‐regulated in miR‐155 inhibitor transfected group, compared with that in NC group (Figure 2B and 3B). Furthermore, partial increased of FOXO3a protein expression was observed in the groups cotreated with rSjP40 and miR‐155 inhibitor, compared to that in the group cotreated with rSjP40 and inhibitor NC (Figure 3B). These results suggest that miR‐155 inhibitors may partially restore rSjP40‐induced down‐regulation of FOXO3a expression.

As biological markers of hepatic fibrosis,^29^ the expression levels of α‐SMA and collagen I were detected by Western blot. Compared with the control group, the protein expression of α‐SMA and collagen I was significantly down‐regulated in rSjP40‐treated group (*P < 0.05, Figure 3C). Compared with the data from the group cotreated with rSjP40 and inhibitor NC, the protein expression of α‐SMA and collagen I was partially increased in rSjP40 and miR‐155 inhibitor cotreated groups (^&^ *P* < 0.05, Figure 3C). Hence, these results in Figure 3C demonstrate that miR‐155 inhibitor can also partially restore rSjP40‐induced down‐regulation of α‐SMA and collagen I expression. However, although the expression of α‐SMA and collagen I was up‐regulated slightly in LX‐2 cells transfected with miR‐155 inhibitor alone, there was no significant statistical difference between miR‐155 inhibitor + rSjP40‐ group and NC inhibitor + rSjP40‐ group (^NS^ *P* > 0.05, Figure 3C). These data above demonstrate that the miR‐155 may involve in the inhibition process of HSC activation induced by rSjP40 in LX‐2 cells and prevent progression of hepatic fibrosis.

### 3.4 rSjP40 induces miR‐155 expression by promoting its promoter expression in HSCs through a STAT5‐dependent mechanism

**Figure 4:**
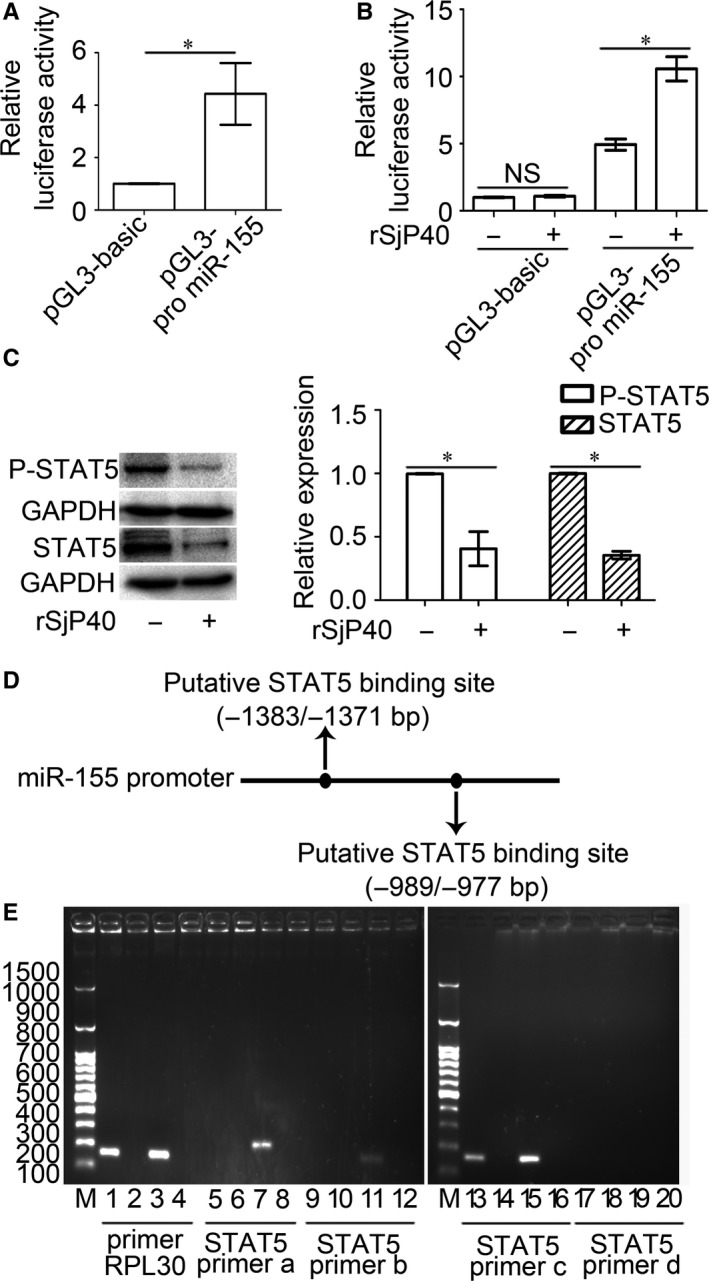
rSjP40‐mediated enhancement of miR‐155 promoter activity is related to STAT5 in LX‐2 cells. A, Luciferase activities of pGL3‐basic and pGL3‐promoter miR‐155 in LX‐2 cells were determined by dual‐luciferase reporter assay. *P < 0.05, compared to pGL3‐basic group. B, The effect of rSjP40 on the luciferase activities of pGL3‐basic or pGL3‐promoter miR‐155 in LX‐2 cells was determined by dual‐luciferase reporter assay. *P < 0.05, compared to each untreated group. C, STAT5 and P‐STAT5 protein expression levels in LX‐2 cells treated with rSjP40 at the concentration of 20 μg/mL were evaluated by Western blot. *P < 0.05, compared to each untreated group. D, Diagram of STAT5 binding sites in the miR‐155 promoter was shown. E, ChIP analysis was performed to confirm the binding of STAT5 to the miR‐155 promoter. M, DL2300 from SMOBIO. Lane 1 represents anti‐Histone H3 group for positive control group. Lanes 5, 9, 13 and 17 represent anti‐STAT5 group for target group. Lanes 2, 6, 10, 14 and 18 represent normal IgG group. Lanes 3, 7, 11, 15 and 19 represent input group. Lanes 4, 8, 12, 16 and 20 represent water group using ddH_2_O as the template for PCR. Primer RPL30 was used for the positive control group

To explore the mechanism by which rSjP40 promotes miR‐155 expression, we constructed a plasmid containing the miR‐155 promoter sequence. Compared with the pGL3‐basic group, the luciferase activity of the miR‐155 promoter group was higher than that of the pGL3‐basic group (Figure 4A). This result confirmed that the promoter sequence of miR‐155 was constructed successfully. Then rSjP40 further promoted the luciferase activity of the miR‐155 promoter, but with no influence on pGL3‐basic (Figure 4B).

Many studies show that there is a close relationship between transcription factors and miRNAs.^30^, ^31^, ^32^ Through the prediction and screening of transcription factor binding sites of miR‐155 promoter via PROMO and UCSC online websites, we speculated that the transcription factor STAT5 may act on the activity of miR‐155 promoter. Western blot results showed that rSjP40 could inhibit both STAT5 and P‐STAT5 protein expression in LX‐2 cells (Figure 4C). According to the results of bioinformatics analysis, two binding sites containing (−1383 bp to −1371 bp) position and (−989 bp to −977 bp) position existed in miR‐155 promoter (Figure 4D). To verify that STAT5 binds directly to miR‐155 promoter, primers (Table 1) were designed for ChIP experiments, and ChIP results showed that STAT5 may bind to the promoter of miR‐155 at the position of −989 bp to −977 bp, subsequently activating miR‐155 transcription (Figure 4E). The above results indicate that rSjP40 can affect miR‐155 promoter activity through STAT5 to induce miR‐155 expression.

## 4 DISCUSSION

Hepatic fibrosis, in which activated HSCs play a central role, is a pathological process of overdeposition of ECM in the liver caused by multiple aetiologies.^33^ In this process, a variety of hepatic damage factors including viral infection, schistosomiasis, alcohol abuse and autoimmune reactions often activate HSCs and subsequently excessive secretion of α‐SMA, collagens, laminin and other ECM proteins from the activated HSCs will result in the formation of hepatic fibrosis.^34^, ^35^ Hence, inhibition of HSC activation and induction of apoptosis and senescence of activated HSCs have become an effective strategy to fight hepatic fibrosis. Schistosomiasis, which is caused by *S. japonicum*, is a kind of parasitic disease that seriously endangers human health, and inflammatory granuloma and hepatic fibrosis are the main pathological characters of Schistosomiasis.^36^, ^37^ Although SEA from schistosomes was considered as the traditional virulence factor, both SEA and its main component P40 from schistosomes (*S. japonicum* and *S. mansoni*) exhibited potential antifibrotic features in HSCs directly.^5^, ^6^, ^7^, ^11^, ^13^, ^38^, ^39^ During this antifibrotic process induced by SEA or its component, whether miRNAs can be involved in is an interesting issue to attract us.

Recently, many miRNAs affecting HSC activation have been identified, suggesting that miRNAs play a potential role in the pathogenesis of hepatic fibrosis.^40^, ^41^, ^42^ For instance, miR‐122, the most abundant miRNA in liver, was down‐regulated significantly during hepatic fibrosis.^43^, ^44^ It has been demonstrated that miR‐29 is negatively correlated with ECM deposition in HSCs and involves an interaction with the PDGF‐mediated signalling pathway.^45^ Lakner et al^46^ showed that high expression of miR‐19b inhibits the TGF‐β signalling pathway in activated HSCs by decreasing TGF‐β receptor II (TGFβRII) expression. In our preliminary studies, we also confirmed some associated miRNAs including miR‐155 in this study may involve in rSjP40‐inhibited HSC activation (Data of miRNAs except miR‐155 are not shown here).

miRNA‐155 is located on the third exon of the B‐cell integration cluster (BIC) gene on human chromosome 21.^47^ As a powerful miRNA, miR‐155 is increased in nonalcoholic fatty liver disease (NAFLD) models and regulates cholesterol and fatty acid metabolism in the liver by targeting LXRα, preventing hepatic steatosis.^48^ Zhang et al^49^ found that miR‐155 plays a key role in the process of alleviating HMGB1‐induced inflammatory effect by soluble CRISPLD2. Dai et al^24^ have also demonstrated that inhibition of miR‐155 expression may induce HSC activation through ERK1 pathway. Which is more important, Cai et al^50^, ^51^ also reported that miR‐155 exhibited a peak in the liver of mice infected with *Schistosoma japonicum* and Hong et al have concluded that miR‐155 may be involved in the regulation of hepatic inflammatory responses and the development of schistosomal hepatopathy. In this study, we observed that miR‐155 expression increased in rSjP40‐treated LX‐2 cells (Figure 1). We further confirmed that miR‐155 involved in rSjP40‐inhibited HSC activation by directly targeting FOXO3a expression (Figure 3).

Studies show that transcription factors often play a messenger role in cells and either promote miRNA or inhibit miRNA transcription. Previously, Gatto et al^52^ demonstrated that transcription factor NF‐κB could bind to miR‐155 promoter at two binding sites and induce miR‐155 expression in Epstein‐Barr virus‐positive B cells. And Yin et al^53^ also reported that the human BIC/miR‐155 genes were transcribed by AP‐1 transcription factor in Epstein‐Barr virus‐transformed lymphoma. Similarly, Marsolier J et al^54^ observed that the transcription factors c‐Jun and AP‐1 induce the expression of miR‐155 in leucocytes transformed by the parasite Theileria. Some transcription factors are also reported to bind to the promoter to inhibit the target gene expression. Recently, He et al^55^ found that transcription factor IRF2 could bind to the promoter of miR‐351 and inhibit its expression to regulate schistosomiasis hepatic fibrosis. Similarly, transcription factors BRCA1, p53, p63 and STAT3 can also inhibit miR‐155 promoter activity in various cells.^56^, ^57^, ^58^ In our study, we also found that transcription factor STAT5 regulates miR‐155 transcription by binding to the position of −989 bp to −977 bp in promoter of miR‐155 (Figure 4E) and rSjP40 could inhibit the expression levels of P‐STAT5 and STAT5.

In conclusion, our results indicate that rSjP40 enhances the expression of miR‐155 by inhibiting the expression of STAT5, thereby inhibiting the expression of FOXO3a and inhibiting the activation of HSCs.

## CONFLICT OF INTEREST

The authors declare that they have no conflict of interest.
